# Audit results of the use of soft cast heel protection devices on patients with heel pressure ulceration

**DOI:** 10.1186/1757-1146-3-S1-P12

**Published:** 2010-12-20

**Authors:** Keri Hutchinson, Ruth Alexander, Scott Cawley

**Affiliations:** 1 Cardiff and Vale University Health board, Cardiff, Wales, UK

## Introduction

There is limited data available in the use of soft casting to offload heel ulceration. Heel pressure ulceration is a costly problem in the health board involved in this study with 15% of in-patients having heel pressure ulceration (HPU).This has led to heel ulcers coming under the 1000 lives campaign in Wales. Soft cast is a quick, cost effective treatment modality for foot ulceration which is largely untested on heel ulceration.

## Methods

12 patients with HPU of grade II and above were issued with a soft cast heel protection device and monitored closely. Size and depth of the wound was recorded as was neurological and vascular status and VAS pain scale scores.

## Results

12 patients were audited, 1 patient died in an unrelated adverse event and of the remaining 11 patients, 2 HPU resolved and the remaining 9 patients ulcers all reduced in size and the wound beds improved. All 11 patients had significantly reduced pain scores with patient stories indicating good compliance and acceptability of the device.

## Discussion

The audit results highlight a need for a research project in this area as the indications were that soft cast heel protection was a successful, cost effective modality for this patient group with the potential for reducing hospital stays and staff time costs.

